# Mass Spectrometry Imaging proves differential absorption profiles of well-characterised permeability markers along the crypt-villus axis

**DOI:** 10.1038/s41598-017-06583-4

**Published:** 2017-07-25

**Authors:** Anna Nilsson, Alexandra Peric, Marie Strimfors, Richard J. A. Goodwin, Martin A. Hayes, Per E. Andrén, Constanze Hilgendorf

**Affiliations:** 1 Science for Life Laboratory, Biomolecular Imaging and Proteomics, National Resource for Mass Spectrometry Imaging, Department of Pharmaceutical Biosciences, Uppsala University, Uppsala, Sweden; 2 Cardiovascular and Metabolic Diseases, Innovative Medicines and Early Development, AstraZeneca Gothenburg, Gothenburg, Sweden; 3 Mass Spectrometry Imaging, Drug Safety and Metabolism, Innovative Medicines and Early Development, AstraZeneca Cambridge, Cambridge, United Kingdom; 4 Safety and ADME Translational Sciences, Drug Safety and Metabolism, Innovative Medicines and Early Development, AstraZeneca Gothenburg, Gothenburg, Sweden

## Abstract

Knowledge about the region-specific absorption profiles from the gastrointestinal tract of orally administered drugs is a critical factor guiding dosage form selection in drug development. We have used a novel approach to study three well-characterized permeability and absorption marker drugs in the intestine. Propranolol and metoprolol (highly permeable compounds) and atenolol (low-moderate permeability compound) were orally co-administered to rats. The site of drug absorption was revealed by high spatial resolution matrix-assisted laser desorption ionization mass spectrometry imaging (MALDI-MSI) and complemented by quantitative measurement of drug concentration in tissue homogenates. MALDI-MSI identified endogenous molecular markers that illustrated the villi structures and confirmed the different absorption sites assigned to histological landmarks for the three drugs. Propranolol and metoprolol showed a rapid absorption and shorter transit distance in contrast to atenolol, which was absorbed more slowly from more distal sites. This study provides novel insights into site specific absorption for each of the compounds along the crypt-villus axis, as well as confirming a proximal-distal absorption gradient along the intestine. The combined analytical approach allowed the quantification and spatial resolution of drug distribution in the intestine and provided experimental evidence for the suggested absorption behaviour of low and highly permeable compounds.

## Introduction

During the development of new therapeutics it is important to build an understanding of the parameters that influence absorption. This can facilitate drug design, allow more accurate pharmacokinetic predictions and influence strategies for drug delivery. Methods to estimate the rate and extent of oral absorption of small molecule drugs have been refined over the past decades^1–4^, and general concepts to predict and classify drug behaviour are well established^5–7^. There are different factors that drive the extent and rate of absorption from the gut, not only the properties of the drug molecule itself, the physiological condition of the patient, but also the dosage form^8–12^. An early understanding of the physicochemical parameters describing the drug molecule, the mechanism of absorption (passive or carrier mediated) and where in the gastrointestinal tract the absorption takes place, enables efficient optimisation in drug design. The site of absorption, in terms of the longitudinal absorption profile of a new drug in the intestine, is a critical factor in developing either modified release formulations which direct the dose to specific regions in the intestine or for sustained release formulations which may require colonic absorption^2, 13, 14^.

To date, less experimental research has been spent on understanding the differences in absorption properties along the crypt-villus axis, in part due to the complexity and scale of intestinal structures. Understanding all dimensions of the site of absorption, longitudinally from duodenum to colon, and vertically from villi tips to crypts, is an important element enabling the prediction of the right dose and the right dosing regimen, but also opens up the possibility for directing drugs to specific targets in the intestinal structure, such as an individual cell type along the crypt villus axis. The *in vitro* method of choice for determining permeability and predicting oral absorption behaviour is the Caco-2 cell monolayer assay. This model is based on a single cell type of columnar epithelial absorptive cells, typically grown on flat monolayer sheets, and as such does not permit studies that investigate the impact of different cell types in the intestine, e.g. mucus-producing goblet cells, or the intestinal structure itself^15, 16^. Different insights into absorption behaviour can be gained by direct measurements using *in vivo* derived samples or incubations with novel microphysiological *in-vitro* systems that comprise multiple cell types and a biorelevant 3D-structure^17, 18^. However, until now, there have been few bioanalytical techniques that allow the direct assessment of compounds in relation to tissue substructures e.g. in the intestinal villi or the submucosa^19^.

Matrix-assisted laser desorption ionization mass spectrometry imaging (MALDI-MSI) allows for simultaneous visualization of administered drugs as well as endogenous compounds such as lipids, peptides, and small proteins^20, 21^. It has increasingly been applied to the determination of biodistribution and quantification of compound in tissue sections^22–29^ and is widely used in drug metabolism and pharmacokinetic (DMPK) research to understand efficacy and toxicity^30–33^. A detailed understanding of absorption processes could be gained by direct MALDI-MSI analysis of intestinal tissue sections at high spatial resolution. Commercially available MALDI-MSI instruments offer lateral spatial resolution at 5 µm without oversampling. These new technical advancements allow researchers to apply MALDI-MSI to address biological or pharmacological questions as the resolution approaches the size of individual cells^34, 35^. In addition to the laser size as instrumental factor influencing the spatial resolution, the matrix crystal size obtained during sample preparation is a factor which can determine resolution.

The aim of this study was to use the high spatial resolution of MALDI-MSI to study differential drug diffusion along the crypt-villus axis as well as longitudinal absorption from proximal to distal small intestine in rats. Tissues were studied to identify endogenous compounds that could serve as specific surrogates for the epithelial lining of the villi, and the underlying submucosal tissue inside the villi. Three well-characterized drugs were selected based on their physicochemical properties and differential permeability behaviour *in-vitro* and *in-vivo*. Propranolol and metoprolol, two highly permeable and well absorbed compounds, and atenolol, a low to moderately permeable compound, (FDA^36^, Table 1) were subjected to MALDI-MSI analysis to determine the site specific absorption in rat small intestine following oral administration. For comparison, liquid chromatography-electrospray ionization tandem mass spectrometry (LC-ESI MS/MS) was performed to quantify the drug concentrations along the proximal to distal segments of the intestine. In addition to the proximal-distal absorption measurements, high spatial resolution MALDI-MSI demonstrated that it was possible to relatively quantify and resolve the drug distribution in the intestinal mucosa and to measure the drugs’ vertical absorption profiles over and along the height of the villi.

**Table 1:** Physicochemical Data, MW, and log D (octanol/water, pH 7.4), and Caco-2 permeability for the three tested drugs (from refs 1, 15 and 48).

Compound	Propranolol	Metoprolol	Atenolol
Molecular weight (g/mol)	259.35	267.37	266.34
LogD (7.4)	1.45	−0.287	−1.51
Caco-2 P_app_ (1E-6.cm/s)	41.9	27.0	0.20
Ion Class	Base	Base	Base
Structure			

## Results

### Defining endogenous villi markers in the intestine by MALDI-MSI

**Figure 1:**
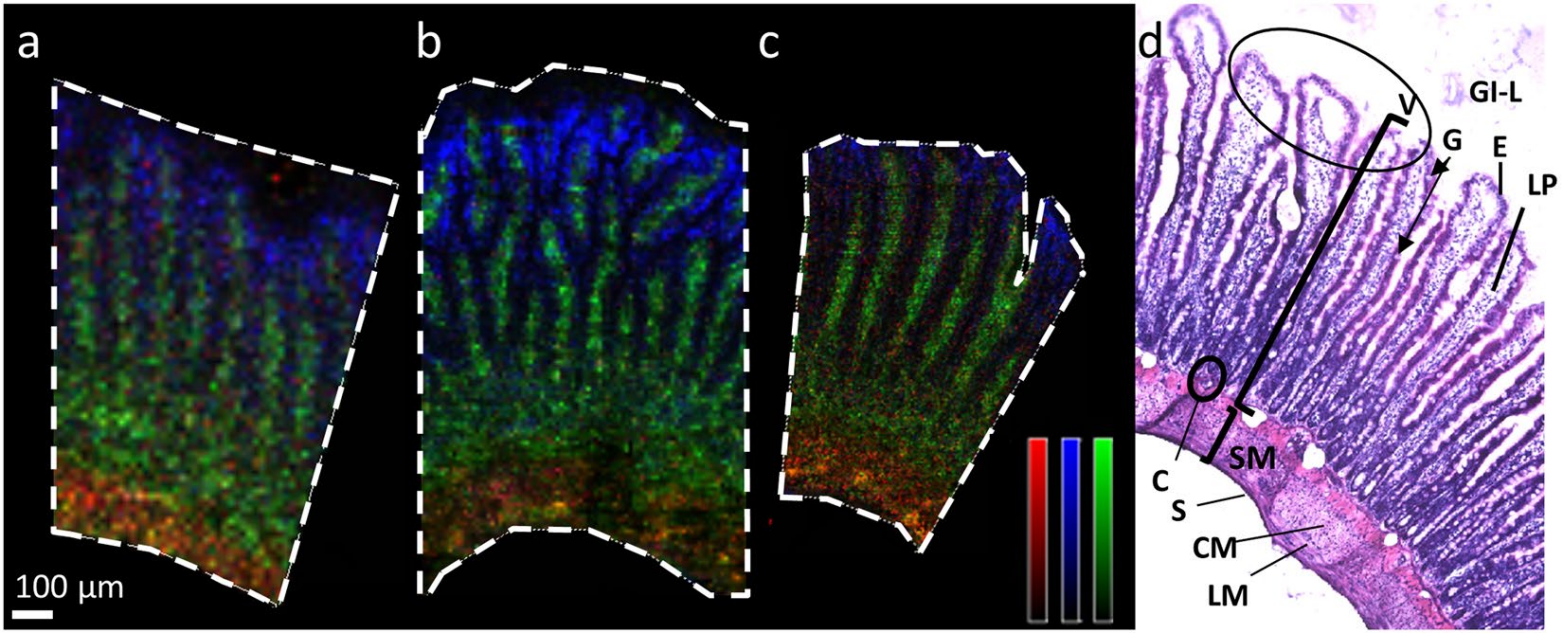
Ion distribution images of three different endogenous compounds. (**a–c**) MALDI MSI ion distribution images of three different endogenous compounds (m/z 772 (green), m/z 769 (red) m/z 804 (blue)) representing different anatomical structures of the intestine lamina propria, submucosa, and epithelial cell layer, respectively. Data was acquired at three different spatial resolutions on three different tissue sections from intestine (**a**) 15 µm, (**b**) 10 µm, and (**c**) 5 µm. The ion intensities are displayed on monochrome colour scales where red is scaled to 50% of max intensity, blue is scaled to 25% of max intensity, and green is scaled to 60% of max intensity. Scale bar 100 µm. (**d**) H&E stained image of fresh frozen rat intestine processed in same way as sections for MSI analysis with annotated regions of the intestine. GI-L = gastrointestinal lumen; V = villi, oval circle villus tips; SM = submucosa; E = epithelial enterocytes (dark eosinophilic stain); G = goblet cell (arrow tip, not stained in H&E); LP = lamina propria; C = crypt base; S = serosa; LM = longitudinal muscle; CM = circular muscle.

Describing compound distribution across the villi of a rat intestine by MALDI-MSI requires an experimental set up where the tissue fixation, MALDI matrix crystal size and the spatial resolution are optimized to enable the fine anatomical features, like the villi structure, to be spatially resolved. An optimized sample work-up process was applied to all subsequent samples, which were analysed at a range of different spatial resolutions (Fig. 1a–c). Three different endogenous compounds were simultaneously detected and selected to distinguish the anatomical structures of the small intestinal mucosa. Each of them is displayed as individual ions (Fig. 1a–c). One endogenous compound’s distribution (*m/z* 772.6, displayed in green) exhibited a high signal in the central region of the villi, associating it to the lamina propria region of the villi. A second (*m/z* 804.6, displayed in blue) followed the outline of the villus protrusions and served to denote the villus epithelium. Finally, a third endogenous compound (*m/z* 769, displayed in red) localized predominantly in the submucosal region. Though unidentified and not representing precise cell-types or microanatomical structures, these endogenous markers enabled the simultaneous co-localization of different areas across the crypt-villus sites and aid visualization of the overall anatomical structures of the intestine using MALDI-MSI. This allowed the villi structures to be recognised by MALDI-MSI and the conclusion that individual villi can be resolved at 15 µm spatial resolution. Greater structural resolution is achieved at 5 µm spatial resolution as it provides a greater number of sampling points across the width of each villus. However, the sample analysis time quadruples every time spatial resolution is increased by a factor of two. Therefore, to balance spatial resolution and analysis time, 15 µm spatial resolution were used for some of the analyses. MSI endogenous marker patterning was alignable to a haematoxylin and eosin (H&E) stained tissue section (Fig. 1d) from a vertical section cut through the mucosa with its villi and the submucosa of the intestine.

### Characterization of site-specific drug absorption in the intestine

**Figure 2:**
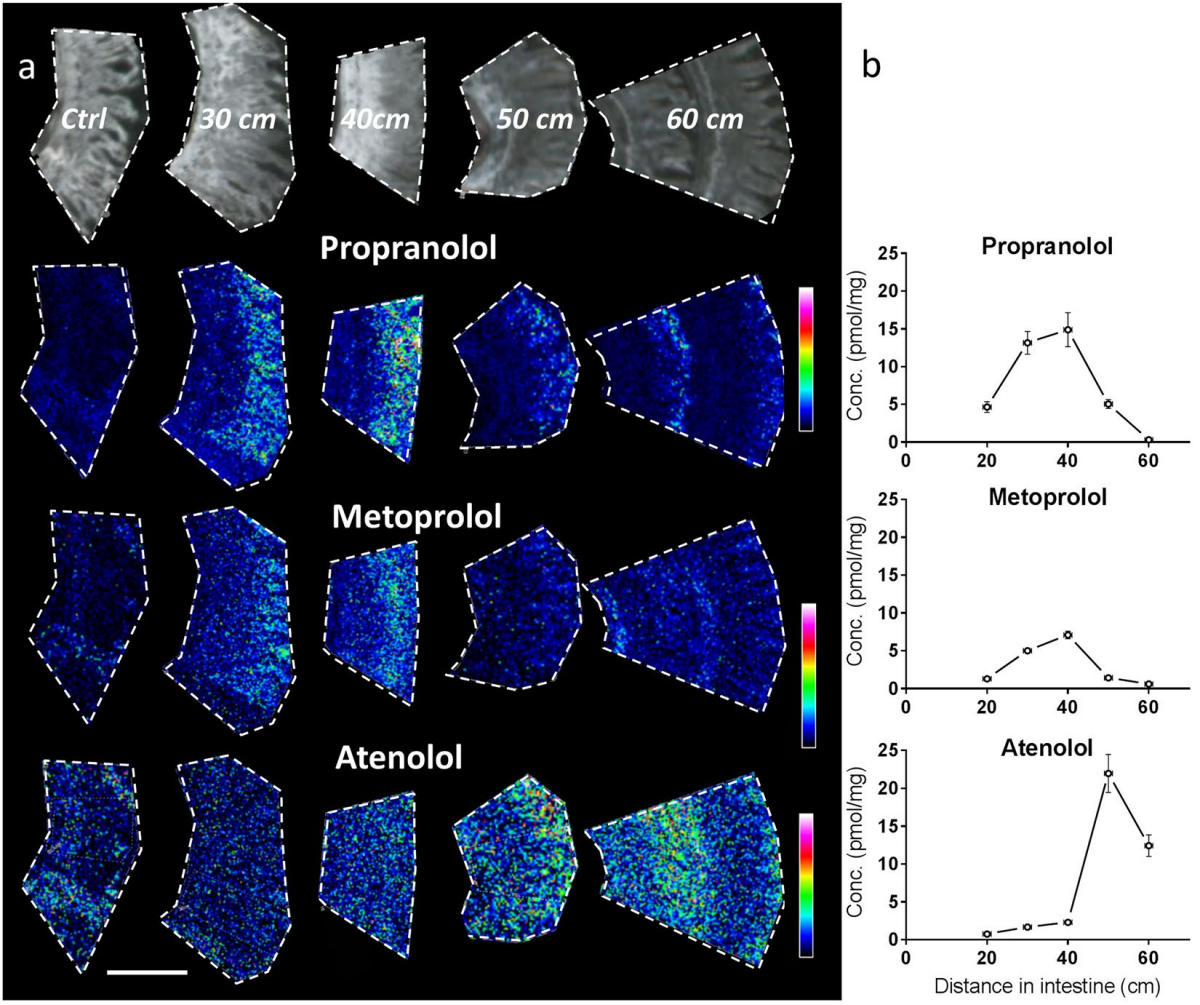
Longitudinal (proximal to distal) and crypt-villus distribution of test compounds in rat small intestine. Images and concentration determinations from samples collected 20 min post oral administration (1 µmol/kg) at different distances distal from the pyloro-duodenal transition. (**a**) MALDI MSI ion distribution images of propranolol, metoprolol, and atenolol in intestine, scale bar is 1 mm. All tissue sections were analysed at the same occasion and each compound is displayed with its own relative intensity scale (rainbow). Propranolol intensity is scaled to 10% of max intensity, metoprolol intensity is scaled to 10% of max intensity, and atenolol intensity is scaled to 7% of max intensity. (**b**) Homogenate concentrations measured by LC-MS/MS for the three investigated compounds; propranolol, metoprolol, and atenolol. The graphs present mean concentrations and range of duplicate determinations, in some instances error bars are shorter than height of symbols. X-error bars indicate position of the sampling +/− 1 cm distal from the pyloro-duodenal transition.

The MALDI-MSI analysis provided details on the relative distribution of each of the administered compounds. At an oral dose of 1 µmol/kg, propranolol and metoprolol showed highest relative abundance around 30–40 cm downstream from the stomach into the small intestine, and had gradually decreased by 50–60 cm. In contrast, atenolol demonstrated highest abundance at 50–60 cm distance from the stomach (Fig. 2a). The ion images acquired at 15 µm spatial resolution show that propranolol and metoprolol were mostly located around the upper part of the villi, while atenolol is more evenly distributed along the whole crypt-villus axis. However, at this resolution we were unable to infer much about the distribution of the compounds into the micro-structures of the villi. This required higher spatial resolution analysis and was performed separately.

To obtain absolute concentrations of the compounds for comparison, adjacent tissue was analysed using a standard bioanalytical method. Hence, compound transit along the intestine and the absorption into the intestinal wall was measured by quantitative LC-ESI MS/MS measurement of intestinal homogenates and compared to MALDI-MSI using matched tissue regions (Fig. 2, Supplementary Fig. S1) for both the 1µmol/kg as well as the 10µmol/kg cassette dose. The abundance of propranolol, metoprolol and atenolol 20 min after 1µmol/kg oral administration varies from the proximal to more distal segments of the small intestine (Fig. 2b). The highest concentrations of propranolol and metoprolol were observed 30 to 40 cm from the pyloro-duodenal junction with decreasing concentration at a distance of 50–60 cm. In contrast, atenolol concentrations were highest in the 50 cm sample. These quantitative measurements match the relative abundance distribution data obtained by MALDI-MSI in adjacent tissue pieces (Fig. 2a).

As anticipated there is a direct relationship between oral dose and maximal tissue concentration, with the 10 µmol/kg dose resulting in ten-fold higher maximal concentrations compared to the 1 µmol/kg dose (Fig. 2, Supplementary Fig. S1). Both LC-MS/MS and MALDI-MSI results were aligned and confirmed this. In contrast to the lower dose both metoprolol and atenolol were detected at the highest concentration in the 20 cm sample 20 min post administration with decreasing levels towards the more distal parts of the jejunum, while propranolol concentration peaked at 30 cm, 20 min post dose.

In addition, higher concentrations of all compounds were measured in the 20 min post-dose samples compared to 45 min post administration (Supplementary Fig. S1). Propranolol and metoprolol concentrations in the tissue samples quantified by LC-MS/MS, consistently showed an almost 3-fold lower concentration at the 45 min time-point compared to the 20 min samples (cf. 40 cm, Supplementary Fig. S1). Propranolol was detected at high levels by MSI in the corresponding ‘40 cm’ sample, and about 3-fold lower levels were measured at the later time point (Supplementary Fig. S1). Similarly to propranolol, relative concentration measurements by MSI showed that intestinal metoprolol decreased 3–4 times from 20 minutes to 45 minutes post dose. Notably, atenolol exposure did not decrease as rapidly as the other two compounds between 20 and 45 min post-dosing, with about 50% of compound remaining in the intestine 45 min post-dosing. The MALDI-MSI also revealed that with the 10 µmol/kg dose (Supplementary Fig. S1) the highest abundance of propranolol and metoprolol was found in the upper villus region, while atenolol showed a more even distribution along the height of the villi.

### High spatial resolution imaging of propranolol, metoprolol and atenolol in the rat jejunum villus structures

**Figure 3:**
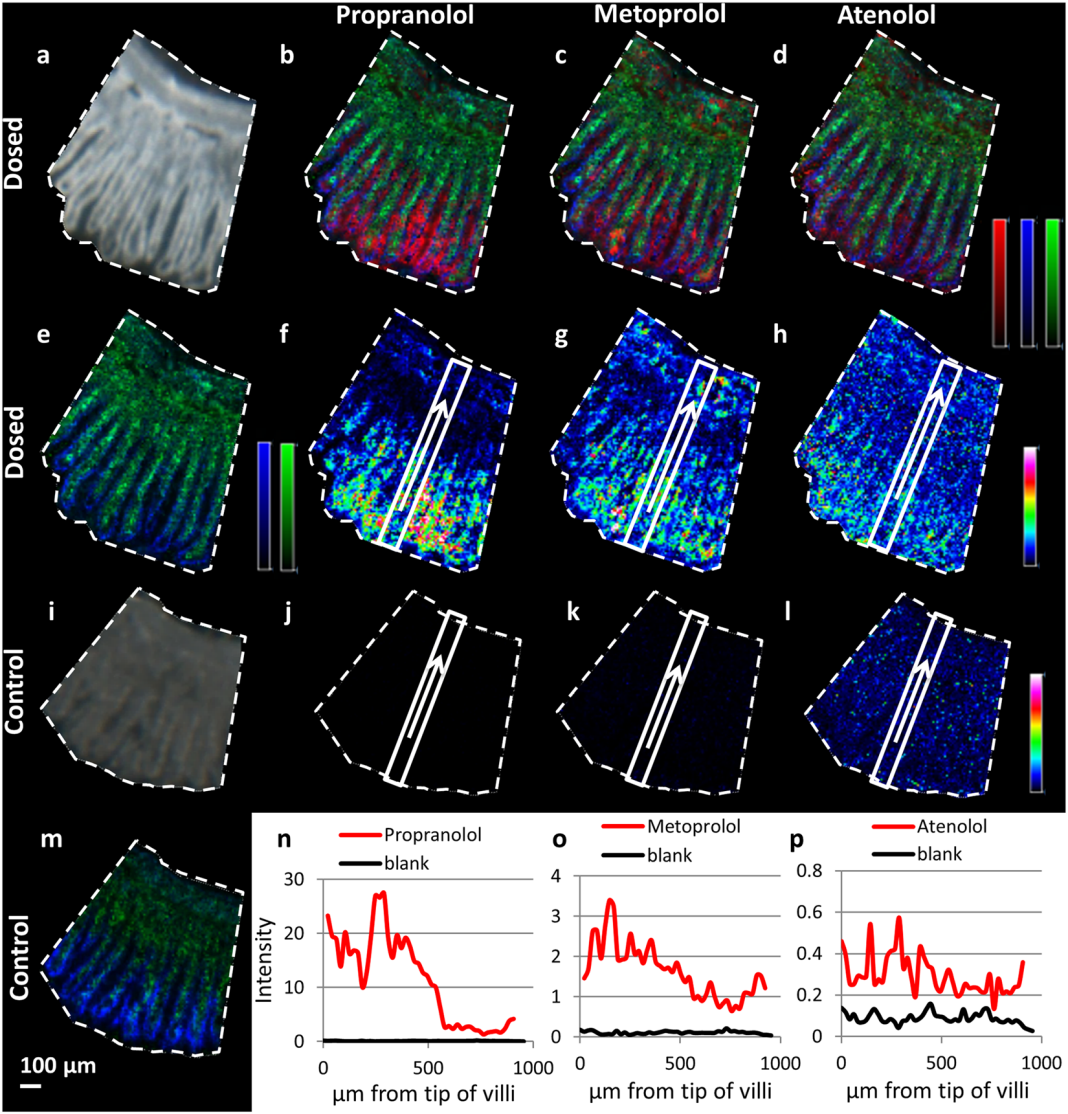
Distribution profiles of the three selected drugs in rat small intestine following oral dosing. (**a**) Scanned photo of tissue 30 cm distal from the pyloro-duodenal junction, collected 20 min post administration (10 µmol/kg). (**b–d**) Ion distribution image of drug (red) together with two endogenous compounds used as villi markers (green m/z 772.6, blue m/z 804.6); (**b**) propranolol (m/z 260), (**c**) metoprolol (m/z 268), (**d**) atenolol (m/z 267). (**e**) Ion distribution image of two villi markers (green m/z 772.6, blue m/z 804.6) on dosed tissue. (**f–h**) Ion distribution image of drug represented by a rainbow colour. The rectangle areas and arrows from villi tips to submucosa mark the area from which the compound abundance is transformed into the line graphs in panels n–p. (**f**) Propranolol (m/z 260), respective line graph red line in panel n. (**g**) metoprolol (m/z 268), respective line graph red line in panel o. (**h**) atenolol (m/z 267) respective line graph red line in panel p. (**i**) Scanned photo of control tissue, not dosed with compound. (**j–l**) Ion distribution images of compounds propranolol (**j**, m/z 260), metoprolol (**k**, m/z 268), atenolol (**l**, m/z 267) with rectangles indicating the area from which the intensity was derived to produce line graphs (blanks in panels n-p respectively). (**m**) Ion distribution image two villi markers (green m/z 772.6, blue m/z 804.6) on control tissue. (**n–p**) line graphs of compound exposure along the crypt-villus axis in dosed (red) and non-dosed (black) tissue, (**n**) propranolol, (**o**) metoprolol, **p**) atenolol. The intensity levels are extracted as average intensity from the tip towards the base of the villi, from the area marked with rectangles in panels f–h (dosed) and (**j–l**) (control). (**b–e**,**m**) are represented by monochrome colour scales; red - scaled to 60% of max intensity, green - scaled to 40% of max intensity, blue - scaled to 30% of max intensity. (**f–h** and **j–l**) are represented by a rainbow colour scaled to 60% of max intensity for each individual m/z. Images were acquired at 10 µm spatial resolution. Scale bar 100 µm.

To investigate in greater detail the vertical distribution of the three compounds along the height of the villi structures, 5 µm spatial resolution images (10 µmol/kg dose, 20 min post administration, 30 cm distance along the intestine) were collected. The increased spatial resolution allowed better visualization of the columnar villi structure from villus tips to crypts and the associated drug distribution over the villus width between epithelium outline and lamina propria (Fig. 3) (villi markers, *m/z* 772.6, green and *m/z* 804.6, blue). However, it is difficult to visually understand the distributions when co-displaying the compound distribution image. Therefore the data was reprocessed using msIQuant^37, 38^ and the intensity for each of the compounds was plotted along the crypt - villus axis (Fig. 3).

The extracted line graph shows that propranolol was detected along the crypt-villus axis from the villus tips to the submucosal region. Propranolol distribution shows a significant reduction in abundance midway down the villi height (Fig. 3n) at approximately 400–500 µm from the villus tips. In the upper villi region abundance is consistently high and then shows a sharp reduction in concentration in the lower regions towards the crypt. Metoprolol has a similar distribution but shows a less steep gradient from the villus tip towards the crypt region over a 200 µm distance. Atenolol levels in the MALDI-MSI maintain similar intensities all the way from the villus tip to the basal regions.

**Figure 4:**
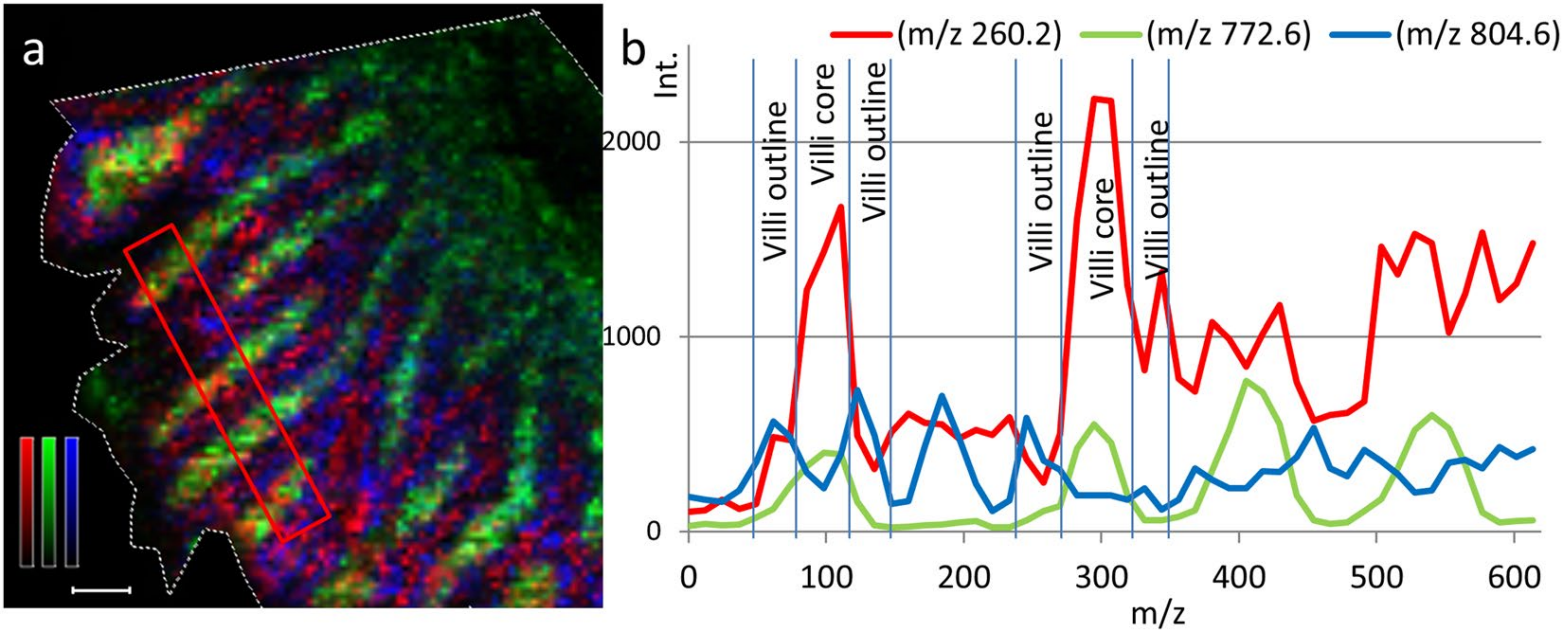
Ion distribution image and transverse profile of propranolol in rat small intestine villi following oral administration. (**a**) Ion distribution images of propranolol (m/z 260.2) together with two different villi markers; m/z 772.6 represents lamina propria region and m/z 804.6 represents the zone of the epithelial cells of the villi outline. Propranolol is represented by a red monochrome colour scale; scaled to 10% of max intensity, m/z 772.6 is represented in green; scaled to 60% of max intensity, and m/z 804.6 is represented in blue; scaled to 20% of max intensity. Scale bar is 100 µm. (**b**) The intensities of propranolol (red), m/z 772.6 (green), and m/z 804.6 (blue) are also represented by line graphs.

To make an assessment of the distribution across and within the villi, the image was reprocessed perpendicularly to the villus axis (Fig. 4). Propranolol displays the highest abundance at the central villus regions. Both endogenous compounds enriched in the central villi region and propranolol have the highest intensities between signal peaks of the marker that follows the epithelial lining. This indicates that propranolol is located preferentially inside the tissue, in the region of the lamina propria. Similar measurements for the two other compounds (Supplementary Fig. S2 and S3) revealed that atenolol is localized primarily between the villi while metoprolol shows a more continuous profile suggesting compound localized both inside the tissue and in between and the villi structures.

## Discussion

The present study demonstrates that MALDI-MSI is a valuable tool for the characterization of the absorption profile in microstructures of the gastrointestinal tract at high spatial resolution using three model compounds with different physiochemical properties. Propranolol and metoprolol are two highly permeable compounds, demonstrating rapid and high fraction absorbed, while atenolol represents a low to moderate permeability compound. Theoretically a high permeability profile would be associated with preferential absorption in the villus tip area, whilst lower permeable, more hydrophilic compounds would utilise a greater permeation area along the crypt villus axis^15, 39–41^.

The obtained tissue profiles are in agreement with published oral pharmacokinetics for the three compounds, where propranolol and metoprolol showed a rapid oral absorption profile with maximum plasma concentration (Tmax) between 10 and 30 min^42, 43^. Both MALDI-MSI and LC-MS analysis clearly demonstrated higher tissue concentrations 20 min after oral dosing than after 45 min, which is consistent with the rapid absorption from the GI-tract and distribution into the systemic circulation.

The low-moderate permeability model drug atenolol is characterised by a different profile. The absorption phase is relatively prolonged and Tmax reached between 1.5 and 3 hours after oral dosing^43^. The present results confirmed a previous report where high concentrations of atenolol were still found in the intestinal tissues 45 min after oral dosing and the peak concentration travelled to more distal regions than for metoprolol and propranolol. It should be noted that these observations are more clearly pronounced at the 1 µmol/kg dose level, whilst at 10 times higher doses regional distribution profiles for all three marker drugs appear more flat, and merely temporal differences with lower GI-concentration after 45 min are observed throughout (Supplementary Fig. S1).

Moreover, a recent publication reemphasized the need to consider the importance of effective and available surface absorption for individual drug molecules^44^. Aligning the theoretical structural and permeability properties of the three model drugs suggests that highly permeable and rapidly absorbed drugs are efficiently absorbed from the upper villus region, whilst soluble drugs with slower membrane permeation would utilise a larger area along the full villus height for permeation^39, 45^. This has been explored in modelling studies^39^ whereas the present work provides *in vivo* experimental evidence for the concept. The images and exposure line plots obtained by MALDI-MSI illustrate the differential disposition profiles of propranolol, metoprolol and atenolol and correlate with their lipophilicity, solubility and permeability properties. Propranolol, the most lipophilic drug, with the highest permeability, exhibits the highest tissue concentrations in samples from the proximal jejunum region. The ion images and the respective line graphs for propranolol confirm the preferred abundance of the compound for the upper villus.

Furthermore, metoprolol is also classified as a highly permeable, rapidly absorbed drug^36^, and the tissue concentration profiles are similar to those of propranolol, with higher GI-tissue concentrations 20 min after dosing than after 45 min and in the proximal jejunal region. Additional insight can be gained from the MS-images, where metoprolol is less restricted in abundance to the upper half of the villi than propranolol. The images and respective line-graph visualisations also show a more even distribution along the crypt-villus axis. Whilst metoprolol is clearly defined and rapidly absorbed with a fraction absorbed above 85% of the dose in humans^46^ and a Tmax in the rat of <20 min, MALDI-MSI highlights that metoprolol differs from propranolol in spatial distribution. Atenolol diffuses and permeates over a larger surface area of the villi than propranolol (Fig. 3f and h). It can be assumed that the differences in lipophilicity and permeability, as measured by logD and Caco-2 Papp-values (Table 1), translate into differences in local disposition along the crypt-villus axis. Atenolol, the most hydrophilic and least permeable test drug has an even abundance profile from the villus tip to the base. This confirms the concept that hydrophilic drugs benefit most from the enhanced surface area of the villi structures of the upper intestine and are able to diffuse between villi, through the hydrophilic mucus and to be absorbed over the entire villi surface.

Visual inspection of the images (Fig. 4 and Supplementary Fig. S2, S3) suggests that at 20 min, propranolol and metoprolol are detected inside and between the villi structures, while atenolol abundance is highest in the intervillus channels. This observation together with the differential abundance plots from villi tips down to the crypt region experimentally confirms the concept previously outlined^39, 41^ that more permeable compounds are mainly absorbed through the villus tips, or the upper part of the intestinal villi.

In conclusion, this study provides the first experimental evidence for the concept that high permeability drugs, associated with rapid oral absorption, are being absorbed through the enterocytes at the villus tips and readily reach the blood stream. High resolution MALDI-MSI of the prototypic marker drug propranolol confirmed this pattern. The highest abundance was found in the upper part of the villi and it accumulated inside the tissue of the villus protrusions. In contrast, the more hydrophilic and less permeable marker atenolol diffused along the intervillus channel, thereby utilising a larger absorption surface area than propranolol. Permeation along an extended length of the small intestine have been demonstrated for atenolol in humans^2^ and rats^47^. This study confirms that atenolol absorption additionally occurs across a larger area of the crypt-villus axis, and contributes to the overall absorption of this drug being around 50%^4^, despite having low permeability (about 50-fold slower permeability than propranolol *in-vitro*, Table 1). The three selected drugs in this study represent prototypic high and low permeability bases. Their MALDI-MSI data, which visualises diffusion along the villus-tip to the basal crypts and their permeation patterns into the villi lamina propria, are in agreement with the postulated and mathematically modelled behaviour of such prototypes. The results from the present study advocate applying this knowledge in drug design to enable local or targeted drug delivery to the intestine and facilitate modelling approaches in biopharmaceutics and dosage form design.

## Methods

All reagents and drugs (Table 1) were purchased from Sigma Aldrich, Germany unless stated otherwise. Water, methanol, formic acid, dihydroxybenzoic acid (DHB) and trifluoroacetic acid (TFA) were obtained from Merck, Germany.

### *In vivo* study and tissue collection

Male rats (Han-Wistar, ~ 100 days old, body-weight ~ 300 g) were used in this study. The animals had free access to food and water and were fasted overnight prior drug administration and sacrifice. All experiments were performed in accordance with relevant guidelines and regulations, the animal work was approved by the Local Animal Research Ethics Board of Gothenburg, Sweden.

The three drugs (atenolol, metoprolol tartrate and propranolol hydrochloride) were dissolved in water and cassette administered to rats *via* gavage at 1 µmol/kg and 10 µmol/kg respectively with a dose volume of 10 mL/kg. The small intestine was collected 20 and 45 min post administration. Four animals were dosed in this study, and non-dosed tissue was prepared from control rats in parallel. The three selected compounds are well characterised drugs and their *in-vivo* pharmacokinetics have been established in many species^4, 42, 43^. Therefore, in the present study we focussed on the concept of “vertical” absorption gradients along the crypt-villous axis, which has not been studied earlier.

The intestine was removed under anaesthesia using isoflurane (Forene, Abbott, USA), to ensure full viability and oxygenation of the tissue until the moment of excision. Animals were sacrificed without regaining consciousness by aortal incision and an overdose of isoflurane. Immediately after excision, intestines were divided into 10 cm long segments and each segment was carefully washed with Krebs-Bicarbonate-Ringer buffer (KBR) from the stomach to the large intestine (additional information in Supplementary Data). The first 5–10 mm of each segment were cut, quickly weighed and collected in homogenization tubes for subsequent extraction and LCMS analysis of total drug concentration in the gut-wall. The next 8–9 cm of each segment were prepared for MALDI-MSI analysis. This was divided into 3 cm long pieces, each opened along the mesenterial border and embedded in a 3% carboxymethyl cellulose (CMC) solution in 0.9% NaCl. The CMC gels quickly on wet-ice/isopropanol and provides tissue support during the subsequent cryosectioning. All samples were stored at −20 °C until cryosectioning.

### Tissue sectioning

The embedded samples were attached and positioned with OCT-compound (TissueTek) to the specimen holder of a microtome (Cryo-Star HM 560 M, Microm International, Germany) set to −20 °C. The samples were sectioned at 10 µm thickness. Each section was thaw-mounted onto an ITO glass slide for MALDI-MSI (237001 Glass Slide, Bruker Daltonics, Germany). A control tissue section from the same intestinal region from non-dosed animal was placed on each slide to enable a direct comparison to be made. All slides were kept at −80 °C until MALDI-MSI analysis. Tissue sections were also stained using haematoxylin and eosin (H&E) following a standard histological protocol for fresh frozen tissues.

### MALDI matrix optimization and preparation

Ionization efficiency tests for the three different test compounds were investigated both on MALDI target and on tissue using four different matrices (α-cyano-4-hydroxycinnamic acid, dihydroxybenzoic acid (DHB), sinapinic acid, and 9-amino acridine). DHB was found to be most efficient MALDI matrix for all compounds and was therefore used for the main study. Additional optimization was performed using a robotic matrix sprayer (TM-Sprayer, HTX Technologies, USA) in order to minimize delocalization and crystal size, while still maintaining adequate extraction of compound from the tissue. Tissue sections on slides were transferred from −80 °C to a desiccator and dried for approximately 30 min prior to matrix coating. An optical image (9600 dpi) of the glass slide was acquired using a flatbed scanner (Epson perfection V500). The tissue sections were coated with DHB (35 mg/ml, 70% ACN, 0.2% TFA) using a robotic sprayer with a flow of rate 70 µl/min, stage speed of 1100 mm/min, track spacing of 2 mm (crisscross pattern), nitrogen pressure 6 psi, and spray nozzle temperature set to 95 °C for 8 passes.

### MALDI-MSI analysis

The MALDI-MSI experiments were carried out in positive reflectron ionization mode over a mass range of *m/z* 120–1020 using two MALDI-TOF/TOF instruments (ultrafleXtreme or rapifleX TissueTyper, Bruker Daltonics) both equipped with Nd:YAG lasers. Data was collected at a spatial resolution of 5 to 15 µm, summing up 300 laser shots at each fixed raster position. FlexImaging 4.0 (Bruker Daltonics) and msIQuant 2.0 was used for data analysis, normalization (to total ion count) and ion image extraction.

### Liquid chromatography tandem mass spectrometry analysis

Tissue homogenates were prepared by tip-sonication in 75% acetonitrile, 1% formic acid, and internal standard (200 nM 5,5-dimethyl-1,3-diphenyl-2-iminobarbituric acid (DDIBA)) using a sonicator (Branson sonifier 250, Branson, USA) and centrifuged at 4000 rpm, 4 °C for 30 min. The supernatants were transferred to fresh vials and centrifuged again under the same conditions. The second supernatant was transferred to a 96-well plate, and diluted with water to a total concentration of 25% acetonitrile, 0.33% formic acid and 67 nM internal standard (DDIBA). The samples were analysed by LC-MS/MS and quantified against a calibration standard curve. The LC-MS/MS analysis was performed using an electrospray ionization tandem quadrupole mass spectrometer (Quattro Premier, Waters, USA) in positive ionization mode. The mass spectrometer was integrated with an ultra-performance liquid chromatography (UPLC) system (Acquity, Waters) assisted with a column manager. Chromatography was performed on a column (Acquity UPLC HSS T3, 2.1 × 50 mm) at 40 °C. The flow rate was 1000 µL/min and the injection volume was 5 µL. The mobile phases consisted of A: water, 0.2% (v/v) formic acid and 2% (v/v) acetonitrile and B: acetonitrile, 0.2% (v/v) formic acid. The linear gradient was as follows: 0.1% B from 0 to 0.3 min, 0.1% to 95% B from 0.3 to 1.3 min, 95% B from 1.3 to 1.6 min, 95% to 0.1% B from 1.6 to 1.61 min, and 0.1% B from 1.61 to 1.80 min. The run time was 1.80 min and the equilibration time between injections was 1 min. The chromatographic front (0.3 min) was diverted to waste. Data was acquired using multiple reaction monitoring (MRM) and mass transitions determined by QuanOptimize (Waters). Instrument control and data processing was performed using MassLynx 4.1 software including QuanLynx (Waters). Concentrations were determined against matrix-spiked calibration standards over a range of 6.8nM- 1 µM, for all methods weighted linear regression (1/X^2^) was applied. The calibration sample residuals showed homoscedastic distribution. Accuracy was < 15% for all three analytes, at the lowest calibration concentration for propranolol and atenolol < 20%. Analytical bias (precision) was ±7% for the propranolol method, ±3.5% for metoprolol and ±8% for atenolol.

## Electronic supplementary material

Supplementary Information 
