# Supplementary material for: Mass Spectrometry Imaging proves differential absorption profiles of well-characterised permeability markers along the crypt-villus axis
Source: Sci Rep. 2017 Jul 25;7:6352. doi: 10.1038/s41598-017-06583-4 (PMC5526999; doi:10.1038/s41598-017-06583-4)
Supplement: Supplementary file 1 — Supplementary Information [file 41598_2017_6583_MOESM1_ESM.pdf]

Mass Spectrometry Imaging proves differential absorption profiles of well-characterised permeability markers along the crypt-villus axis

Supplementary Information

Anna Nilsson[1,+], Alexandra Peric[2,+], Marie Strimfors[2], Richard J.A. Goodwin[3], Martin A. Hayes[2], Per E. Andrén[1, ¥] and Constanze Hilgendorf[4, ¥, *]

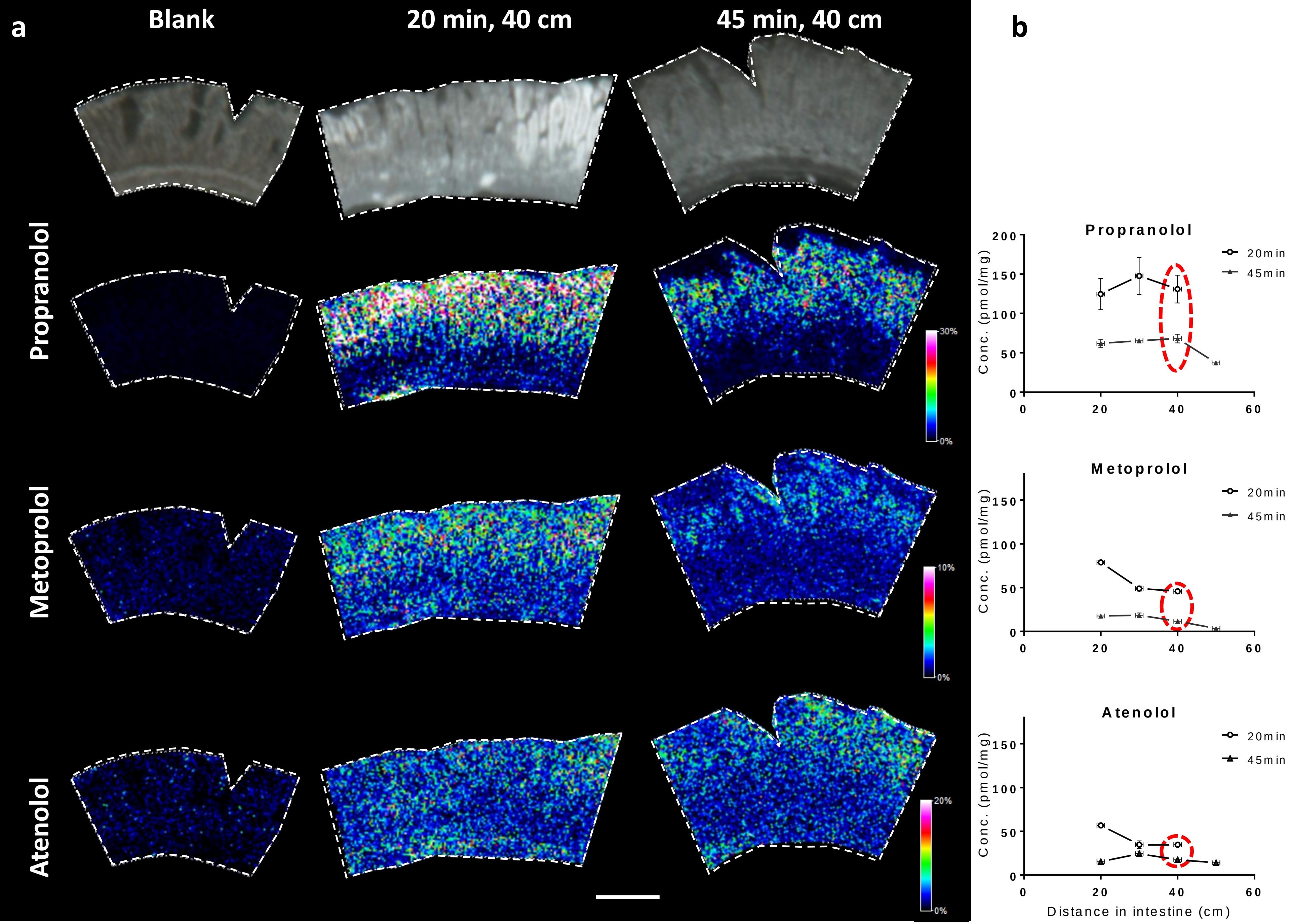

a
Blank
20 min, 40 cm
45 min, 40 cm
Propranolol
Metoprolol
Atenolol
30%
0%
10%
0%
20%
0%
b
Propranolol
Metoprolol
Atenolol
20min
45min
Conc. (pmol/mg)
Distance in intestine (cm)

Supplementary Figure S1

Temporal distribution of test compounds in rat small intestine; representative concentration determinations at 40 cm from the pyloro-duodenal transition and adjacent MALDI-MS-images, 20 min and 45 min post oral administration (10 µmol/kg).

a) MSI ion distribution images of propranolol, metoprolol, and atenolol in intestine. All tissue sections were analysed at the same occasion, and each compound is displayed with its own relative intensity scale (rainbow). Propranolol intensity is scaled to 30% of max intensity, metoprolol intensity is scaled to 10% of max intensity, and atenolol intensity is scaled to 20% of max intensity. Scale bar is 500 µm.

b) Homogenate concentrations for the three investigated test compounds at two different time points (20 min and 45 min) and at different distances from the pyloro-duodenal transition. The concentration of propranolol, metoprolol, and atenolol was measured by LC-MS/MS. Graphs present mean concentrations and SD, in some instances error bars are shorter than height of symbols. The measurements circled in red represent tissue concentrations adjacent to the intestinal section of the MSI data presented in panel A.

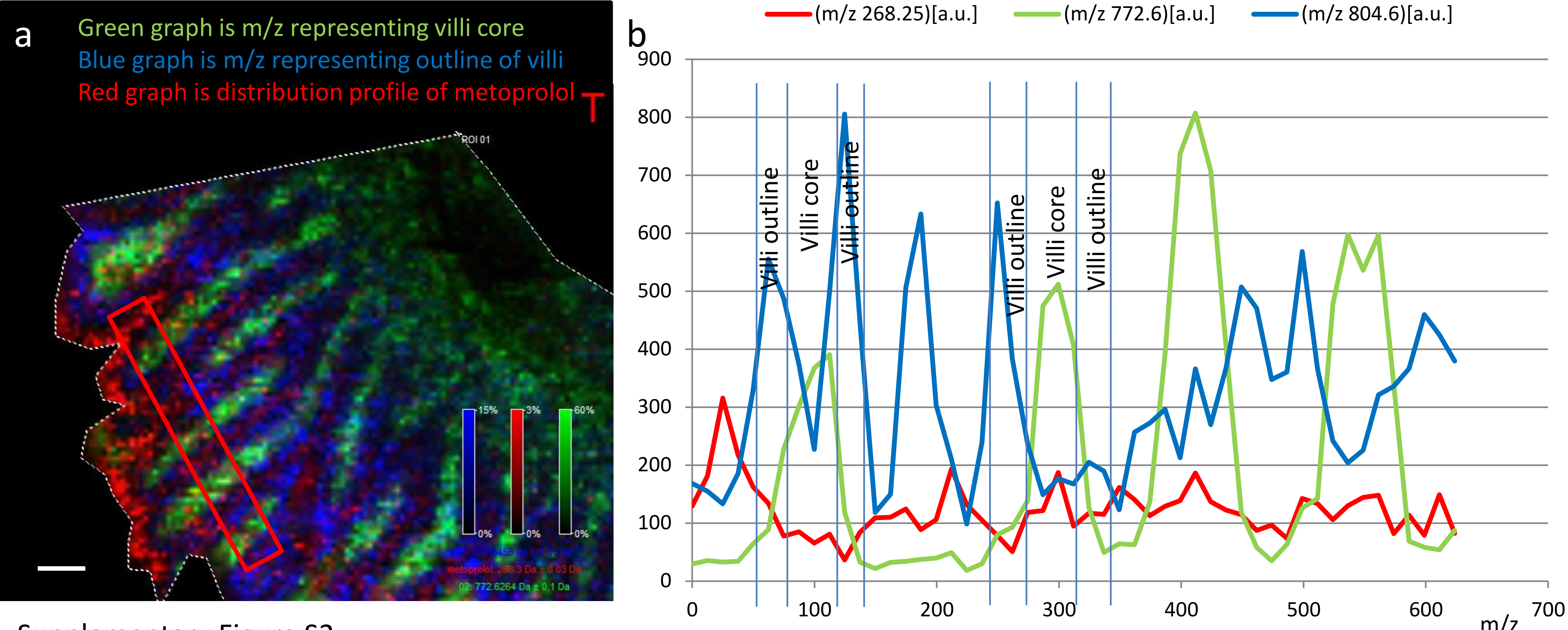

Supplementary Figure S2

Metoprolol distribution profile transverse villi. a) Ion distribution images of metoprolol (m/z 268) together with two different villi markers; m/z 772 represents lamina propria region and m/z 804 represents the zone of the epithelial cells of the villi outline. Metoprolol is represented by a red monochrome colour scale; scaled to 3% of max intensity, m/z 772 is represented in green; scaled to 60% of max intensity, and m/z 804 is represented in blue; scaled to 15% of max intensity. Scale bar is 100 µm. b) The intensities of metoprolol (red), m/z 772 (green), and m/z 804 (blue) are also represented by line graphs.

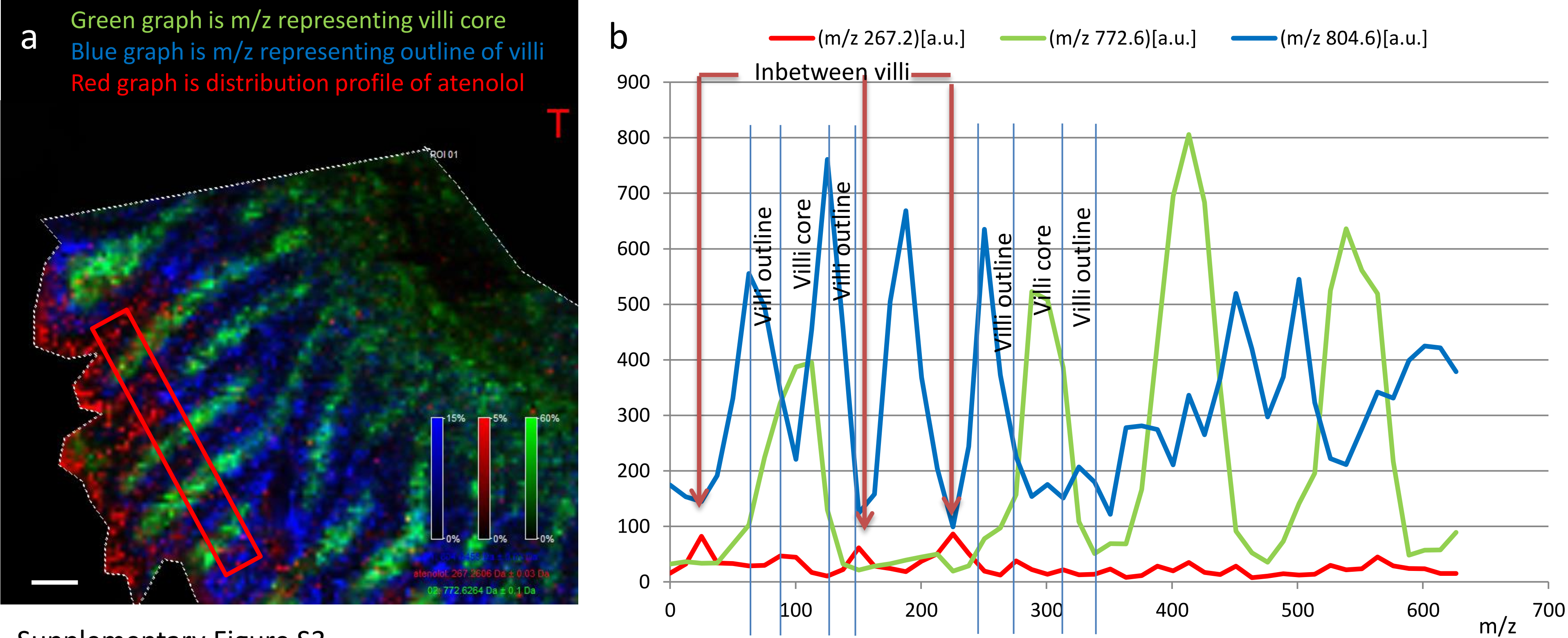

Supplementary Figure S3

Atenolol distribution profile transverse villi. a) Ion distribution images of atenolol (m/z 267) together with two different villi markers; m/z 772 represents lamina propria region and m/z 804 represents the zone of the epithelial cells of the villi outline. Atenolol is represented by a red monochrome colour scale; scaled to 5% of max intensity, m/z 772 is represented in green; scaled to 60% of max intensity, and m/z 804 is represented in blue; scaled to 15% of max intensity. Scale bar is 100 µm. b) The intensities of propranolol (red), m/z 772 (green), and m/z 804 (blue) are also depicted by line graphs.

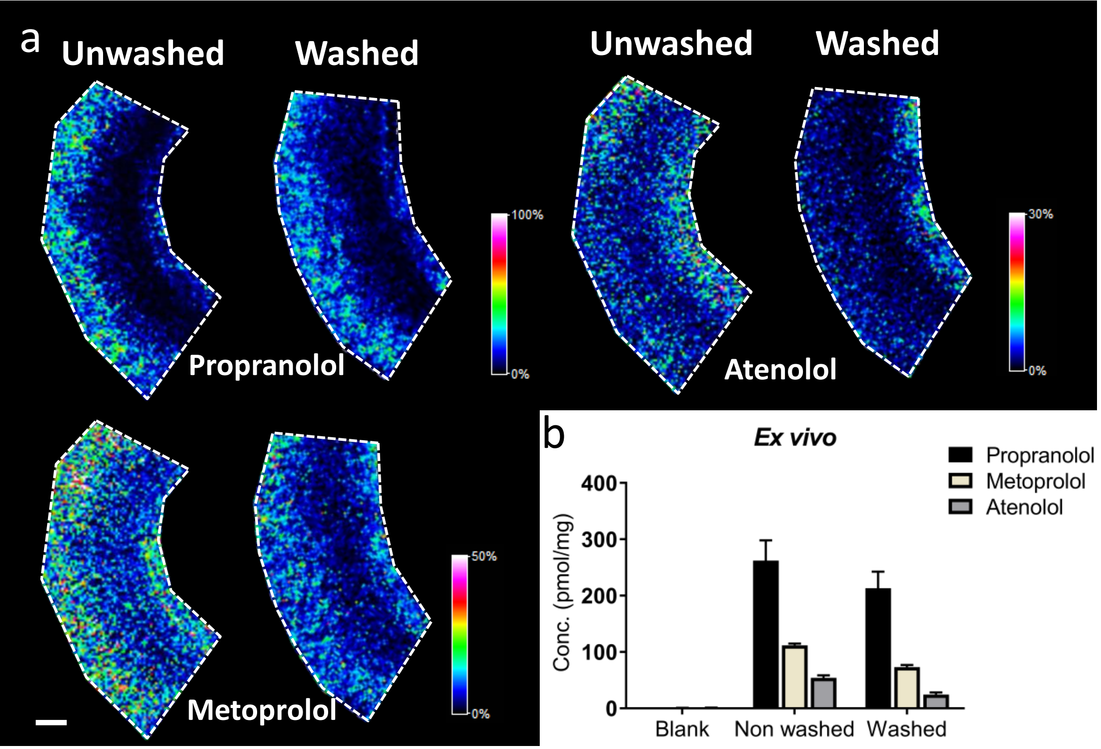

Supplementary Figure S4.

***Ex vivo* absorption and tissue washing analysis.** a) Ion distribution images of propranolol, metoprolol, and atenolol. Dosing was performed ex vivo during a 20 min incubation. Furthermore, the tissue was either directly embedded or quickly washed in Krebs-Bicarbonate-Ringer buffer before embedding the subsequent MALDI MSI analysis. All three compound distributions are presented as a rainbow scale where propranolol is scaled to 100 % of max intensity, metoprolol is scaled to 50 % of max intensity, and atenolol is scaled to 30 % of max intensity. Scale bar is 200µm. b) Quantification by LC MS/MS of propranolol, metoprolol, and atenolol in washed and non-washed tissue. Bar graphs represent mean and CV of duplicate determinations.

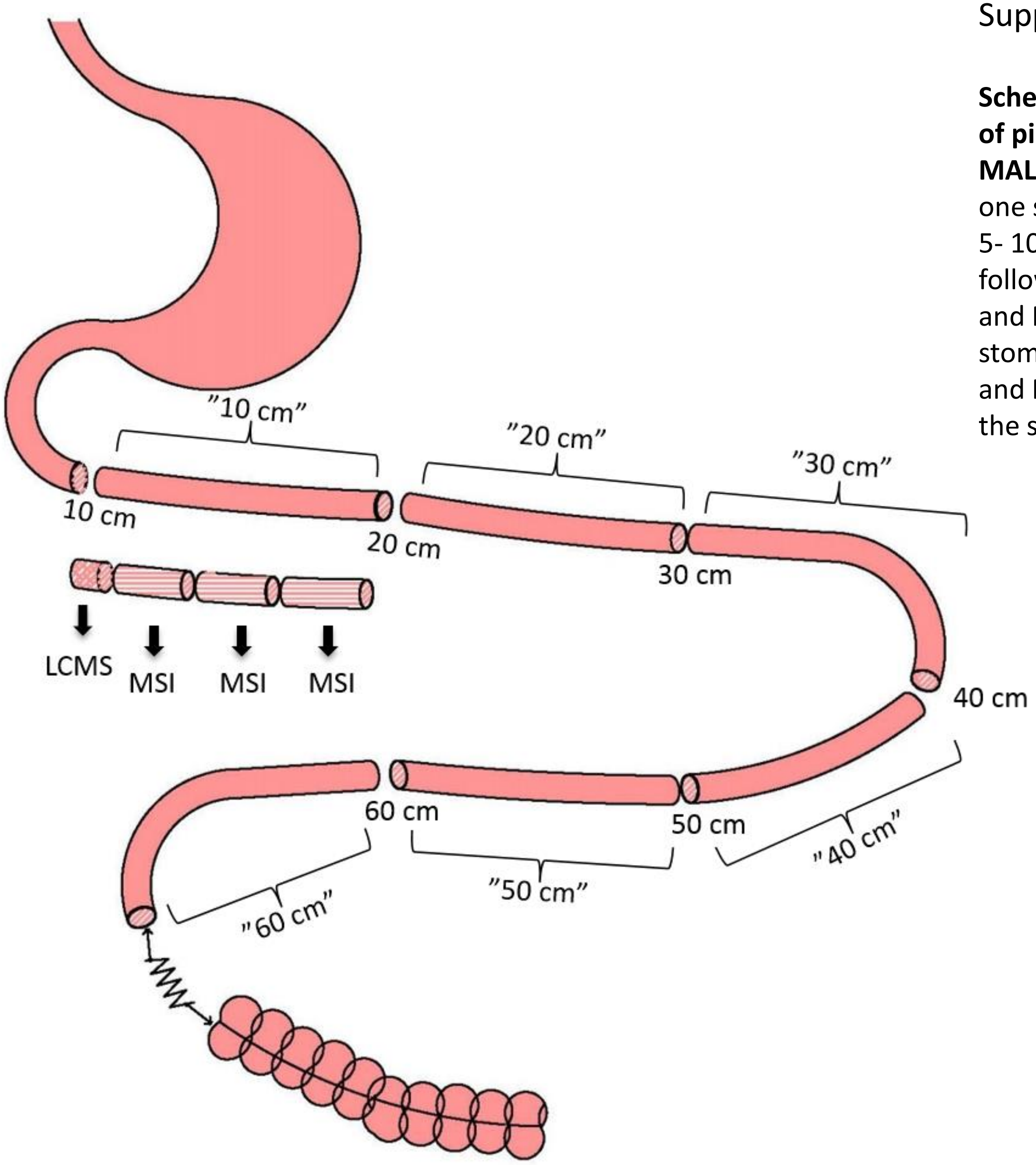

Supplementary Figure S5.

Schematic picture of small intestinal tissue preparation of pieces for homogenisation (LC-MS/MS) and slices for MALDI- MSI analysis. Segments of 10 cm were cut. Within one segment, four sub-segments were cut. The first one, 5- 10 mm were prepared for LC-MS/MS analysis. The three following pieces of 2-3 cm each were prepared for slicing and MSI analysis. First cut was done 10 cm down from the stomach and referred to as "10cm" for both LC-MS/MS and MALDI-MSI results. The next segment, cut 20 cm from the stomach, is referred to as "20 cm" etc.

Supplementary Data and Discussion

Experiments were performed *ex vivo* to determine the impact of the washing of the intestinal lumen on distribution of compounds when analysed by MALDI-MSI. To ensure equal exposure of the tissue segment to all three drugs, independent of transit time and differential absorption properties, a 20 min incubation with 100 µM of each compound was performed in *ex vivo* upper rat jejunum. Tissues were either rapidly flushed with cold saline or not directly opened prior to, tissue embedding, sectioning and MSI analysis at 15 µm resolution. The results from this analysis (Supplementary Figure S4) indicated that the distribution of the beta-blockers in the *ex vivo* study was similar to that detected in tissue following oral dosing (Figure 2). However, following washing of the *ex vivo* jejunum sample there was a notable change in the abundance of test drug, particular for atenolol. Atenolol is the most hydrophilic and least permeable compound and appeared to be reduced from the tissue sample towards the limit of detection. Metoprolol distribution remained similar but with a decreased abundance. Propranolol appeared to be the least affected by washing, with both abundance and distribution appearing to be consistent with unwashed sample. While the purpose of the experiment was to determine optimal sample processing, the results continue to indicate that metoprolol and propranolol may be permeating quickly into the tissue and resulting in higher abundance as detected by MSI analysis and confirmed with homogenization and quantitation. In contrast, atenolol a compound with lower permeability, was seen to have lower concentrations both *in vivo* and *ex vivo*. Atenolol was at lowest abundance following washing and is likely to result from the unabsorbed excess atenolol being readily washed off from the intestinal lumen and tissue samples during the quick flushing procedure.
